# Exploring variations in gait patterns and joint motion characteristics in school-aged children across different walking speeds: a comprehensive motion analysis study

**DOI:** 10.25122/jml-2023-0110

**Published:** 2023-06

**Authors:** Mohd Arshad Bari, Haq Nawaz Mir, Junaid Ahmad Parrey, Amir Ateeq, Arish Ajhar, Wafa Hashem Al Muslem, Shibili Nuhmani, Anas Alduhishy, Mohammed Essa Alsubaiei

**Affiliations:** 1 Department of Physical Education, Aligarh Muslim University, Aligarh, India; 2 Jawaharlal Nehru Medical College and Hospital, Aligarh Muslim University, Aligarh, India; 3 Department of Physical Therapy, College of Applied Medical Sciences, Imam Abdulrahman Bin Faisal University, Dammam, Kingdom of Saudi Arabia

**Keywords:** gait, biomechanical analysis, school-aged children, pediatrics, mobility

## Abstract

This study aimed to investigate differences in gait patterns among individuals with different walking speeds and identify the range of motion (ROM) and angular velocity for various joints during gait. Forty-five schoolchildren were randomly selected for this study. To capture their walking patterns, two FDR-AX700 4K HDR camcorders were positioned to observe the predetermined walkway. Each participant completed a 5-meter walk at various speeds, including slow, normal, and fast, while maintaining a straight stride. There were significantly higher ROM and angular velocity (p<0.05) at the hip, knee, and ankle joints across most stages of walking at a faster speed compared to slow and normal speeds. At the same time, the angular velocity was significantly higher at the hip joint during hip extension terminal stance at normal speed compared to slow and fast speeds (p<0.05, ƞ^2^ =0.74). Similarly, the ROM of knee flexion swing, ankle plantar flexion loading response, and ankle dorsiflexion midswing angular velocity were significantly higher during normal walking speed (p<0.05). Conversely, slow-speed walking showed significantly higher ROM at knee extension terminal swing (ƞ^2^=0.52) and ankle dorsiflexion terminal stance (ƞ^2^=0.78) (p<0.05). The results indicate that individuals with different walking speeds exhibit significant differences in gait patterns. Slower walking speeds resulted in lower gait velocity and different joint motions compared to faster walking speeds.

## INTRODUCTION

Walking is an essential and fundamental activity humans engage in daily activities. However, the biomechanics of walking can vary from person to person, and even small deviations from the norm can lead to gait abnormalities, resulting in pain, injury, and reduced mobility [1-3]. The ability to walk at different speeds is an important aspect of gait, and the biomechanical analysis of gait at different speeds can provide insights into the mechanisms underlying human walking.

Biomechanical gait analysis measures kinematic and kinetic variables such as joint angles, angular velocity, ground reaction force, and muscle activity [4]. Examining these variables provides valuable insights into the fundamental mechanisms of walking. The ability to walk at different speeds is a significant component of gait, and it is crucial to comprehend how speed affects the biomechanics of walking. Various speeds necessitate modifications in the kinematic and kinetic variables of walking, such as stride length, stride frequency, and joint angles [5-7]. Therefore, examining the biomechanics of gait at different speeds is critical for comprehending the impact of speed on walking patterns [8, 9].

School-aged children walk at different speeds depending on various factors such as distance, urgency, and fatigue [10]. Walking speeds can be categorized into slow, normal, and fast, representing different degrees of exertion and energy expenditure. The effects of walking at different speeds on gait biomechanics in school-aged children have not been extensively studied. Therefore, this research aimed to contribute to the knowledge gap by analyzing gait biomechanics in school-aged children at different walking speeds.

Gait analysis in school-aged children can provide valuable insights into their development and help identify any potential problems in their gait patterns [11, 12]. Children in this age group undergo significant musculoskeletal changes, so their walking patterns can be influenced [13]. Biomechanical gait analysis can help identify abnormalities in gait development and provide early interventions that can improve walking patterns and prevent musculoskeletal injuries.

School-aged children represent a unique population because they are in a critical stage of growth and development [14, 15]. It is important to study their walking patterns and the effects of walking at different speeds to identify potential issues in their gait development. The effects of walking at different speeds on the biomechanics of gait in school-aged children have not been extensively studied, and the existing studies have produced inconsistent results. Therefore, this research aimed to conduct a comprehensive biomechanical analysis of gain in school-aged children, focusing on investigating differences in the biomechanics of walking at various speeds.

## MATERIAL AND METHODS

### Participants

A sample of 45 male school-aged children was randomly selected from secondary schools in the Poonch district of Jammu and Kashmir, India. The selection of school-going children as participants in this study was carefully considered by the researcher. Firstly, the choice of male school-going children aimed to ensure consistency and eliminate potential confounding factors associated with gender differences in gait patterns. Secondly, the decision to select participants from secondary schools in the Poonch district of Jammu and Kashmir, India, was based on convenience and accessibility while creating a relatively homogeneous sample regarding the cultural background and environmental factors.

All participants met the inclusion criteria of understanding instructions and cooperating during the assessment. To ensure the validity of the results, some exclusion criteria were also implemented. Children with a history of illness or injury, major surgery, gross biomechanical abnormalities, neurological conditions, comorbidities, or intellectual disorders were not included in the study. By implementing these exclusion criteria, the researchers aimed to minimize potential confounding factors and produce more reliable and accurate results.

### Dropout stabilization facilitators

In this section, particular attention is given to the factors that contribute to the stabilization of dropout, like circumstances that result in the permanent discontinuation of education. These influential factors are elaborated upon in this section, with detailed explanations in Table 3.

### Experimental setup

In this study, two FDR-AX700 4K HDR Camcorder video cameras were synchronized to capture the subject's walking gait actions in a defined, specific walkway. The cameras were securely mounted on tripods and fixed to the floor. To ensure the most accurate reconstruction of the two-dimensional coordinates, the cameras were positioned so that their optical axes intersected perpendicularly on both the sagittal and frontal planes.

Specifically, each camera was situated with its optical axis perpendicular to the sagittal plane and parallel to the mediolateral axis on the side the subject was walking. This arrangement resulted in a 90-degree angle between each optical axis. Two cameras were placed around the walkway: one in the frontal plane, positioned 5 meters behind the starting position, and the other positioned 8.5 meters from the midpoint of the calibrated walking line/axis in the frontal plane, perpendicular to the sagittal plane and parallel to the mediolateral axis on the participants' right side, resulting in an approximately 90-degree angle between their respective optical axes.

The visual field of view of the camera covered a 5-meter walkway, with space provided beyond both ends for starting and finishing steps. For each trial, the cameras captured at least one complete gait cycle within their field of view. The video cameras were set to a sampling rate of sixty (60) frames per second in sports mode to capture moving subjects. Additionally, the shutter speed of the camera was set to a quick speed of 1/2000 to ensure optimal performance in capturing the subject's movement. These technical specifications were critical to collecting accurate data and ensuring the reliability of the results.

### Procedure

The study involved participants who engaged in a series of walking trials to achieve one successful trial for each of the three distinct walking conditions. To meet this goal, each participant was required to complete a maximum of three walking trials on a 5 m walkway. The successful completion of a trial was based on the requirement that it was recorded in the middle of the walkway and captured at least one complete gait cycle or stride without any acceleration or deceleration steps.

To ensure accuracy in the collected data and visibility of reflective markings on the lower limbs and pelvis, participants were instructed to wear tight-fitting shorts during the assessment. This step was necessary to facilitate the researchers' ability to collect precise and reliable data. It is important to note that using informed consent and appropriate attire for the participants is essential in maintaining ethical standards and promoting the safety and well-being of those involved in the study.

The walking trials were conducted under three different walking conditions: normal (N), slow (S), and fast (F) walking. Participants were instructed to walk at their typical comfortable walking speed for the normal walking condition. For the slower walking condition, they were instructed to walk at approximately half of their habitual walking speed, and for the fast-walking condition, they were told to walk at their maximum speed. To capture data from a range of walking speeds, participants could choose their walking speeds for slow, normal, and fast walking situations.

Testing for each participant proceeded in the same order, with slowed walks following normal walking and fast walking. The peak sagittal plane joint angles and angular velocities of the hip, knee, and ankle were then computed to analyze the participants' gait patterns. By conducting the trials under distinct walking conditions, the researchers collected data that accurately represented the participants' walking abilities under different circumstances. This approach was crucial in obtaining reliable results and drawing valid conclusions from the study.

### Data Processing and Analysis

In order to conduct a kinematic analysis of gait, the identified clips of each subject were segmented using specialized software tools such as Silicon Coach Pro 8 (SCP) and Max Traq 3D motion analysis software. Motion analysis software calculated joint angles using captured marker data by defining joint models and establishing marker relationships. Subsequently, angular velocity was derived by differentiating the joint angles over time, with the software offering tools to compute the derivative and obtain angular velocity values.

Descriptive statistics were utilized to examine the participants' demographics, joint angles, and angular velocities for each walking speed increment. Statistical analysis was performed using version 20 of the Statistical Package for Social Science (SPSS-20) software. Mean and standard deviation calculations were used to create descriptive analyses, while the Shapiro-Wilk test was employed to verify the normality of the data (p>0.05). ANOVA was employed to investigate the variations between variables and groups, and Post Hoc (LSD) was used to assess significant mean differences. The criterion for significance was set at 0.05, and the results were analyzed accordingly.

## RESULTS

TThe study sample consisted of 45 school-aged children with a mean age of 17.24±0.73 years, mean weight of 60.74±6.56 kg, and mean height of 173.02±7.06 cm. Table 1 provides a tabular representation of the average trace of sagittal plane joint angles and angular velocities throughout the gait cycle at the hip, knee, and ankle joints at each walking speed (slow, normal, and fast).

**Table 1:** The kinematics of lower limb sagittal plane joints for slow, normal, and fast walking gaits

	Variables	Slow walking (M±D)	Normal walking (M±D)	Fast walking (M±D)
**Range of motion (^0^)**	Gait Velocity (m/s)	0.89±0.60	1.20±0.10	1.53±0.74
	Hip Flexion Loading Response (^o^)	33.54±1.68	30.30±1.68	35.56±1.06
	Hip Extension Pre-swing (^o^)	3.8±.12	8.02±0.54	13.14±0.60
	Hip Flexion Terminal Swing (^o^)	33.68±1.79	33.05±1.30	39.54±3.29
	Knee Flexion Loading Response (^o^)	12.78±1.51	18.54±0.82	24.07±2.25
	Knee Extension Terminal Stance (^o^)	5.58±0.23	5.45±0.81	3.54±0.26
	Knee Flexion Swing (^o^)	70.47±5.59	68.09±3.11	70.50±3.10
	Knee Extension Terminal Swing (^o^)	5.59±0.23	4.27±0.44	5.54±0.87
	Ankle Plantar flexion Loading Response (^o^)	4.9±0.44	4.14±0.51	3.50±0.25
	Ankle Dorsiflexion Terminal Stance (^o^)	15.49±0.81	13.00±0.50	13.01±0.52
	Ankle Plantar flexion Pre-swing	15.43±0.97	21.51±0.90	23.07±1.50
	Ankle Dorsiflexion Mid-Swing (^o^)	6.45±0.82	5.92±0.62	5.77±0.73
**Angular velocity (°/s)**	Hip Extension Loading Response (^o^/s)	90.70±5.72	135.24±2.23	165.28±6.58
	Hip Flexion Pre-swing (^o^/s)	165.91±9.28	211.21±3.39	231.14±6.20
	Hip Extension Terminal Swing	23.98±1.10	30.19±1.92	25.12±1.69
	Knee Flexion Loading Response (^o^/s)	72.41±4.80	138.47±4.08	154.65±6.76
	Knee Extension Terminal Stance (^o^/s)	49.18±1.80	84.79±3.24	107.90±4.47
	Knee Flexion Swing (^o^/s)	276.42±8.55	316.75±3.40	340.33±3.34
	Knee Extension Terminal Swing (^o^/s)	276.91±9.013	352.65±5.03	360.85±6.10
	Ankle Plantarflexion Loading Response (^o^/s)	80.00±5.87	96.25±5.08	118.73±4.25
	Ankle Dorsiflexion Mid-Stance (^o^/s)	197.75±912.67	93.21±3.75	100.07±3.25
	Ankle Plantarflexion Pre-swing	240.33±6.70	324.89±3.97	328.66±4.13
	Ankle Dorsiflexion Mid-Swing (^o^/s)	146.27±26.92	199.98±2.84	209.69±3.08
	Cadence (s/m)	94.38±7.40	107.54±24.48	127.31±7.18

Tables 2, 3, and 4 present the results of one-way analysis of variance (ANOVA) conducted to compare the differences in the range of motion (ROM) and angular velocities at the hip, knee, and ankle joints during different walking speeds (slow, normal, and fast). The ANOVA analysis revealed significant differences in ROM and angular velocities among the three walking speeds at various phases of gait. The post hoc analysis reported significantly higher scores in ROM and angular velocity (p<0.05) at the hip (hip flexion loading response (^o^), hip extension pre-swing (^o^), hip flexion terminal swing (^o^), hip extension loading response (^o^/s) hip flexion pre-swing (^o^/s), knee (knee flexion loading response (^o^), knee extension terminal stance (^o^), knee extension terminal stance (^o^/s), knee flexion loading response (^o^/s), knee flexion swing (^o^/s), knee extension terminal swing (^o^/s)) and ankle (ankle plantar flexion pre-swing (^o^), ankle plantar flexion loading response (^o^/s), ankle plantar-flexion pre-swing (^o^/s), ankle dorsiflexion mid-stance (^o^/s), ankle dorsiflexion mid-swing (^o^/s)) during faster walking speeds compared to slow and normal walking speeds. At the same time, the angular velocity was significantly higher at the hip joint during hip extension terminal stance at normal speed compared to slow and fast speeds (p<0.05). Similar results were observed in ROM of knee flexion swing, ankle plantar flexion loading response, and ankle dorsiflexion midswing angular velocity, where normal walking speed showed significantly higher scores (p<0.05) compared to slow and fast speeds. Furthermore, slow speed demonstrated significantly higher ROM at knee extension terminal swing and ankle dorsiflexion terminal stance (p<0.05).

**Table 2:** Results of one-way analysis of variance (ANOVA) for hip response during walking gait at different speeds

ANOVA								
	Variables		Sum of Squares	Mean Square	F (2,132)	ƞ2	Sig	Post-Hoc
**Range of motion (^0^)**	Hip Flexion Loading Response (^o^)	Between Groups	634.12	317.06	140.24	0.68	0.00*	S<N<F
		Within Groups	298.41	2.26				
		Total	932.53					
	Hip Extension Pre Swing (^o^)	Between Groups	1977.85	988.92	4.33	0.98	0.00*	N<S<F
		Within Groups	30.15	0.22				
		Total	2008.00					
	Hip Flexion Terminal Swing (^o^)	Between Groups	1156.49	578.24	109.91	0.62	0.00*	N<S<F
		Within Groups	694.43	5.26				
		Total	1850.93					
**Angular velocity** **(^0^/s)**	Hip Extension Loading Response (^o^/s)	Between Groups	126744.48	63372.24	2.34	0.97	0.00*	S<N<F
		Within Groups	3571.39	27.06				
		Total	130315.87					
	Hip Flexion Pre Swing (^o^/s)	Between Groups	100576.57	50288.29	1.11	0.94	0.00*	S<N<F
		Within Groups	5989.07	45.37				
		Total	106565.64					
	Hip Extension Terminal Stance (^o^/s)	Between Groups	985.06	492.53	190.07	0.74	0.00*	S<F<N
		Within Groups	342.06	2.59				
		Total	1327.11					

S= slow walking, N=normal walking, F= fast walking, *significant at 0.05 level

**Table 3:** One-way analysis of variance (ANOVA) for knee response at walking gait among different speed variations

ANOVA								
	Variables		Sum of Squares	Mean Square	F (2,132)	ƞ2	Sig	Post-Hoc
**Range of motion (^0^)**	Knee Flexion Loading Response (^o^)	Between Groups	2876.15	1438.07	534.46	0.89	0.00*	S<N<F
		Within Groups	355.17	2.69				
		Total	3231.32					
	Knee Extension Terminal Stance (^o^)	Between Groups	81.96	40.98	87.01	0.57	0.00*	N>S>F
		Within Groups	62.17	0.47				
		Total	144.13					
	Knee Flexion Swing (^o^)	Between Groups	171.58	85.79	5.10	0.07	0.00*	F<S<N
		Within Groups	2221.58	16.83				
		Total	2393.16					
	Knee Extension Terminal Swing (^o^)	Between Groups	49.80	24.90	73.18	0.52	0.00*	N<F<S
		Within Groups	44.91	0.34				
		Total	94.72					
**Angular velocity** **(^0^/s)**	Knee Extension Terminal Stance (^o^/s)	Between Groups	78745.02	39372.51	3.49	0.98	0.00*	S<N<F
		Within Groups	1487.58	11.27				
		Total	80232.60					
	Knee Flexion Loading Response (^o^/s)	Between Groups	170856.51	85428.25	3.00	0.98	0.00*	S<N<F
		Within Groups	3755.80	28.45				
		Total	174612.30					
	Knee Flexion Swing (^o^/s)	Between Groups	93995.66	46997.83	1.47	0.96	0.00*	S<N<F
		Within Groups	4221.33	31.98				
		Total	98216.99					
	Knee Extension Terminal Swing (^o^/s)	Between Groups	192776.29	96388.15	2.01	0.97	0.00*	S<N<F
		Within Groups	6326.58	47.93				
			199102.88					

S= slow walking, N=normal walking, F= fast walking, *significant at 0.05 level

**Table 4:** One-way analysis of variance (ANOVA) for ankle response at walking gait among different speed variations

ANOVA								
	Variables		Sum of Squares	Mean Square	F (2,132)	ƞ2	Sig	Post-Hoc
Range of motion (^0^)	Ankle Plantar Flexion Loading Response (^o^)	Between Groups	40.70	20.35	115.63	0.64	0.00*	F<S<N
		Within Groups	23.23	0.18				
		Total	63.92					
	Ankle Dorsi-Flexion Terminal Stance (^o^)	Between Groups	184.93	92.46	230.88	0.78	0.00*	N>F>S
		Within Groups	52.86	0.40				
		Total	237.79	733.89				
	Ankle Plantar Flexion Pre-Swing (^o^)	Between Groups	1467.77	1.35	545.35	0.89	0.00*	S<N<F
		Within Groups	177.63					
		Total	1645.41					
	Ankle Dorsi Flexion Mid Swing (^o^)	Between Groups	11.56	5.78	10.77	0.14	0.00*	F<S<N
		Within Groups	70.85	0.54				
		Total	82.41					
Angular velocity (^0^/s)	Ankle Plantar Flexion Loading Response (^o^/s)	Between Groups	34063.92	17031.9	651.88	0.91	0.00*	S<N<F
		Within Groups	3448.81	26.13				
		Total	37512.73					
	Ankle Plantar-Flexion Pre-Swing (^o^/s)	Between Groups	224500.77	112250.38	4.33	0.98	0.00*	S<N<F
		Within Groups	3425.10	25.95				
		Total	227925.86					
	Ankle Dorsi-Flexion Mid-Stance (^o^/s)	Between Groups	307784.62	153892.1	0.55	0.01	0.58	S<N<F
		Within Groups	36651644.2	277663.7				
		Total						
	Ankle Dorsi-Flexion Mid-Swing (^o^/s)	Between Groups	105026.23	52513.12	212.14	76	0.00*	S<N<F
		Within Groups	32675.33	247.54				
		Total	137701.56					

S= slow walking, N=normal walking, F= fast walking, *significant at 0.05 level

Table 5 presents the results of the one-way analysis of variance (ANOVA) conducted to assess the impact of different walking speeds on cadence during walking gait. The analysis revealed a significant effect on cadence among different variations of walking speed, with F (2,132)=52.55, p<0 .00, η^2^=0.44. Post-hoc tests were conducted, which showed that the mean step rate for the slow walking gait (M=94.38, SD=8.76) was significantly lower than that for the normal walking gait (M=107.54, SD=10.11), and the fast walking gait (M=127.31, SD=7.44). Additionally, the mean step rate for the normal walking gait was significantly lower than that for the fast walking gait. These findings suggest that step rate is influenced by variations in walking speed, with the slowest walking gait leading to the lowest step rate and the fast walking gait leading to the highest step rate. The effect of walking speed variation was statistically significant, accounting for 44% of the variance in step rate. Overall, these results indicate that walking speed variation significantly impacts step rate, with slower walking speeds leading to slower step rates and faster walking speeds leading to faster step rates.

**Table 5:** One-way analysis of variance (ANOVA) for cadence at walking gait among different speed variations

ANOVA							
Variables		Sum of Squares	Mean Square	F (2,132)	ƞ2	Sig	Post-Hoc
**Cadence** **(s/m)**	Between Groups	12365.1	12365.1	52.55*	0.44	0.00*	S<N<F
	Within Groups	235.31	235.31				
	Total	55792.90					

S= slow walking, N=normal walking, F= fast walking, *significant at 0.05 level

## DISCUSSION

The study aimed to measure gait velocity and joint angles in a small sample of participants under slow, normal, and fast walking conditions. The results indicated that gait velocity increased as walking speed increased, and there were variations in joint angles across the different walking conditions. It is difficult to compare the findings of this study with previous research without knowing their specific details. However, based on the available information, it can be noted that the gait velocity during normal walking in the current study (1.20 ±0.10 m/s) is consistent with the findings of previous studies that have reported a range of 1.14-1.22 m/s for healthy subjects [16]. The gait velocity during slow walking (0.89±0.60 m/s) is lower than the average gait velocity reported for healthy adults [17-19].

Regarding the range of motion, the current study showed that the range of motion for hip flexion loading response was higher during fast walking (35.56±1.06) as compared to slow walking (33.54±1.68) and normal walking (30.30±1.68). Similarly, the range of motion for knee flexion loading response was higher during fast walking (24.07±2.25) as compared to slow walking (12.78±1.51) and normal walking (18.54±0.82). These findings are consistent with previous research that reported a higher range of motion during fast walking [20-22].

The results showed six different variables related to hip flexion and extension during various phases of gait at three different walking speeds: slow, normal, and fast. The analysis revealed that hip flexion loading response was highest for fast walking, followed by normal walking, and lowest for slow walking. This finding is consistent with previous research by Romkes and Bracht-Schweizer [23], which found that walking speed significantly affects hip flexion loading response during gait. However, the mean score for hip extension pre-swing was highest for fast walking, followed by slow walking, and lowest for normal walking. This finding contradicts previous research by Perry and Davids [24], which generally found that hip extension pre-swing increases with walking speed. Similar to the results for hip flexion loading response, the mean score for hip flexion terminal swing was highest for fast walking, followed by normal walking, and lowest for slow walking. This finding is consistent with previous research by Neptune, *et al*. [25], which found that hip flexion during terminal swing increases with walking speed.

The mean score for hip extension loading response was highest for normal walking, followed by slow walking, and lowest for fast walking. This differs from previous research by Perry and Davids [24] and Neumann [26], who found that hip extension loading response increases with walking speed. Similarly, for hip flexion pre-swing, the mean score was highest for normal walking, followed by slow walking, and lowest for fast walking. This finding also differs from previous research by Perry and Davids [24], who found that hip flexion pre-swing decreases with increasing walking speed. Finally, for the hip extension terminal stance, the analysis indicated a statistically significant difference between the mean scores for slow, normal, and fast walking speeds. Specifically, the mean score for hip extension terminal stance was highest for slow walking, followed by fast walking, and lowest for normal walking. This finding is consistent with previous research by Winter [27], who found that hip extension during terminal stance increases with decreasing walking speed.

Table 3 presents the results of ANOVA for the knee flexion and extension range of motion during different gait phases among three groups of participants. The results showed significant differences in knee joint range of motion between the groups in all four gait phases (p<0.05). Kadaba *et al*. [28] found that knee joint motion during gait varied significantly among individuals. They reported that the range of motion in the sagittal plane during the stance phase ranged from 5 to 75 degrees among individuals. Additionally, they found that the peak knee flexion during the swing phase varied between 45 and 80 degrees among participants. Previous studies have consistently demonstrated substantial variations in knee joint motion during walking among healthy individuals. The results of the current investigation further support and confirm the presence of significant differences in knee joint mobility across various walking gaits with different speed variations. The examination and treatment of gait abnormalities as well as the development of prosthetic devices that replicate natural joint motion during walking are all significantly impacted by these results.

This study showed significant differences in ankle joint motion between the different variations of walking gait in all four gait phases (p<0.05). Previous research also investigated ankle joint motion during gait among healthy individuals. Winter *et al*. [29] found that ankle joint motion during the stance phase varied significantly among individuals. They reported that the ankle joint angle at initial contact ranged from -5 to 25 degrees among participants. Additionally, they found that the peak ankle plantarflexion during push-off ranged from 10 to 50 degrees among participants. Another study by Inman *et al*. [30] investigated the effects of different walking speeds on ankle joint motion during gait. They found that increased walking speeds increased ankle plantar flexion and dorsiflexion angles. They also reported that the ankle joint range of motion during the stance phase was larger than during the swing phase. Previous research has demonstrated significant variations in ankle joint motion during gait among healthy individuals. The results in the current study also support the presence of significant differences in ankle joint motion among different variations of the speed (groups) of individuals. These findings have important implications for assessing and treating gait disorders, as well as the design of prosthetic devices that mimic normal joint motion during gait.

In terms of angular velocity, the current study showed that the angular velocity of hip flexion pre-swing, knee flexion swing, and ankle plantar flexion pre-swing was higher during fast walking than normal and slow walking. These findings are consistent with previous studies that reported higher angular velocity during fast walking [19]. The Hip Extension Loading Response variable differed significantly among the three walking gait speeds (S<N<F), with the highest average score observed during fast walking. This result aligns with earlier studies indicating that individuals using flexible prosthetic feet exhibited greater ankle range of motion and a more natural gait pattern [31].

The Hip Flexion Pre-Swing variable showed significant differences among the three walking gait speeds (S<F<N), with the highest mean score observed during fast walking. This finding is consistent with previous research showing that individuals with flexible prosthetic feet display a more natural gait pattern characterized by an increased hip flexion angle during the swing phase [31]. Similarly, The Hip Extension Terminal Stance variable showed significant differences among the three-speed variations (S<F<N), with the slow walking gait having the lowest mean score. This result aligns with a previous study indicating that individuals with a solid ankle-foot prosthesis exhibit a more extended hip during the stance phase compared to those with a flexible foot [32, 33].

The Knee Extension Terminal Stance variable showed significant differences among the three variations of walking gait speed (S<N<F), with fast walking having the highest mean score. This finding is consistent with previous research that found individuals with flexible prosthetic feet have greater knee flexion during the swing phase and greater knee extension during the stance phase [31]. The Knee Flexion Loading Response variable showed significant differences among the three different walking gait speeds (S<N<F), with the fast-walking gait having the highest mean score. This finding is consistent with previous research that found individuals with flexible prosthetic feet have a more normal gait pattern, including greater knee flexion during the loading response phase [31]. The Knee Flexion Swing variable showed significant differences among the three different walking gait speeds (S<N<F), with the fast walking gait having the highest mean score. This finding is consistent with a previous study that found that individuals with a flexible foot had a more normal gait pattern, including greater knee flexion during the swing phase[33]. The Knee Extension Terminal Swing variable showed significant differences among the three different walking gait speeds (S<N<F), with the fast walking gait having the highest mean score, consistent with previous research where individuals with flexible prosthetic feet had greater knee extension during the stance phase and greater knee flexion during the swing phase [31].

The Ankle Plantar Flexion Loading Response variable showed significant differences among the three different walking gait speeds (S<N<F), with the fast walking gait having the highest mean score, consistent with previous research where individuals with flexible prosthetic feet had greater ankle plantar flexion during the loading response phase [31]. The variable of Ankle Plantar-Flexion Pre-Swing showed significant differences among the three different walking gait speeds (S<N<F), with the fast walking gait having the highest mean score. This finding is consistent with previous research, which found that individuals with a flexible foot had a more normal gait pattern, including greater ankle plantar flexion during the pre-swing phase[33]. The results showed a significant difference among the three different walking gait speed for ankle plantar-flexion pre-swing, with the fast walking gait having the highest mean score. This finding is consistent with a previous study that found individuals with a flexible foot had a more normal gait pattern, including greater ankle plantar flexion during the pre-swing phase. In contrast, the results of the study showed no significant difference among the three different walking gait speed for ankle dorsiflexion mid-stance (F=0.55, p=0.58). This finding is inconsistent with previous research, which found that individuals with a flexible foot have a greater range of motion in the ankle and a more natural gait pattern [31]. The results showed a significant difference among the three different walking gait speed for ankle dorsiflexion mid-swing, with the fast walking gait having the highest mean score. This finding is consistent with previous research that found individuals with a flexible foot had a more normal gait pattern, including greater ankle dorsiflexion during the mid-swing phase[33]. These findings have significant implications for both clinical practice and prosthetic design. Clinicians should consider the influence of walking speed when evaluating gait abnormalities, as gait velocity and joint angles exhibit variations under different walking conditions. To create prosthetic devices that emulate natural joint motion, it is crucial to incorporate adaptability to these variations and consider the range of motion in the hip, knee, and ankle joints.

However, this study had certain limitations. Firstly, the sample size was small, which could potentially constrain the generalizability of the results. Secondly, the study was limited to a specific age range, and the findings may not be applicable to other age groups. Thirdly, the study only evaluated gait velocity and joint angles in slow, normal, and fast walking conditions, which may not represent all possible walking conditions. Fourthly, there was a possibility of measurement errors due to the utilization of specific instruments or subjective assessments by the evaluators. Lastly, the study did not include a control group for comparison, restricting the ability to draw conclusions about the observed differences. To overcome these limitations, future research endeavors should incorporate control groups to facilitate meaningful comparisons and yield more robust conclusions. By addressing these methodological constraints, a more comprehensive understanding of gait patterns can be attained, enhancing our knowledge of their implications in clinical practice and prosthetic design.

## CONCLUSION

The study found significant differences in gait patterns among individuals with different walking speeds. The gait velocity during normal walking was consistent with previous research on healthy subjects, but the gait velocity during slow walking was lower than the average gait velocity reported for healthy adults. The range of motion for hip flexion and knee flexion loading response was higher during fast walking than slow and normal walking. The results indicate that hip flexion loading response and hip flexion terminal swing increase with walking speed, while hip extension terminal stance increases with decreasing walking speed. The knee joint range of motion during gait varied significantly among individuals, and the ankle joint motion during gait also varied significantly among individuals with different walking speeds. The findings of this research study highlight the significance of considering walking speed and variations in joint angles when examining gait abnormalities, offering valuable insights for tailoring treatment plans, developing evidence-based interventions, and enhancing patient outcomes in clinical practice and prosthetic design. It is imperative to conduct further research to explore the relationships between diverse walking patterns, joint angles, and varying speeds among different populations. This research will contribute to advancing personalized treatment approaches and improvements in prosthetic design and ultimately lead to improved clinical outcomes for individuals with walking abnormalities.

## Personal thanks

We would like to thank the Department of Physical Education, Aligarh Muslim University, for their invaluable support and guidance throughout the research.
